# Porous Hybrid Nanofibers Comprising ZnSe/CoSe₂/Carbon with Uniformly Distributed Pores as Anodes for High-Performance Sodium-Ion Batteries

**DOI:** 10.3390/nano9101362

**Published:** 2019-09-23

**Authors:** Sun Young Jeong, Jung Sang Cho

**Affiliations:** Department of Engineering Chemistry, Chungbuk National University, Chungbuk 361-763, Korea

**Keywords:** ZnSe, CoSe_2_, porous nanostructure, sodium-ion batteries, electrospinning

## Abstract

Well-designed porous structured bimetallic ZnSe/CoSe₂/carbon composite nanofibers with uniformly distributed pores were prepared as anodes for sodium-ion batteries by electrospinning and subsequent simple heat-treatment processes. Size-controlled polystyrene (PS) nanobeads in the electrospinning solution played a key role in the formation and uniform distribution of pores in the nanofiber structure, after the removal of selected PS nanobeads during the heat-treatment process. The porous ZnSe/CoSe₂/C composite nanofibers were able to release severe mechanical stress/strain during discharge–charge cycles, introduce larger contact area between the active materials and the electrolyte, and provide more active sites during cycling. The discharge capacity of porous ZnSe/CoSe_2_/C composite nanofibers at the 10,000th cycle was 297 mA h g^−1^, and the capacity retention measured from the second cycle was 81%. The final rate capacities of porous ZnSe/CoSe_2_/C composite nanofibers were 438, 377, 367, 348, 335, 323, and 303 mA h g^−1^ at current densities of 0.1, 0.5, 1, 3, 5, 7, and 10 A g^−1^, respectively. At the higher current densities of 10, 20, and 30 A g^−1^, the final rate capacities were 310, 222, and 141 mA h g^−1^, respectively.

## 1. Introduction

Recently, sodium-ion batteries (SIBs) have attracted much attention as next generation energy storage devices due to the abundance and lower cost of sodium resources compared to those of lithium [1,2,3,4]. However, the larger ionic radius and molar mass of the Na ion, compared with those of the Li ion, severely impede overall SIB performance, causing issues such as slow and irreversible Na ion insertion and extraction during cycles. To solve these problems, various metal compounds with sophisticated morphologies have been studied for adoption as SIB anode materials. Among them, well-designed metal selenides, which possess a narrow bandgap and an electrical conductivity higher than that of metal oxides and sulfides, have attracted attention [5,6,7,8].

In particular, it is well established that bimetallic compounds normally exhibit better electrochemical activity and higher chemical and thermal stability than single metal compounds, due to the complex composition, and interfacial and cooperative effects of multiple metal active sites [9,10,11,12]. However, as with other metal compounds, bimetallic compounds also show insufficient electrical conductivity, and suffer from structural expansion, pulverization, and aggregation during repeated charge–discharge processes, which causes mechanical fracture, electrical contact loss, and an unstable solid electrolyte interphase (SEI). Therefore, their practical application in SIBs has not fulfilled expectations, mainly due to poor cycle and rate properties, which greatly hinder use of these active materials in SIBs.

To solve these issues, nanostructuring has proved to be an effective way forward, as nanostructured anode materials share a large contact area with the electrolyte, possess short Na ion and electron pathways, and can accommodate the strain associated with repeated cycles [13,14,15,16]. Therefore, it has been suggested that a suitable nanostructure for anode materials could effectively improve SIB Na ion storage properties.

In this study, we introduced well-designed porous structured bimetallic ZnSe/CoSe₂/carbon composite nanofibers (PZCN), with uniformly distributed pores, prepared by electrospinning and subsequent simple heat-treatment processes. The size-controlled polystyrene (PS) nanobeads in the electrospinning solution played a key role in the formation and uniform distribution of pores in the nanofiber structure, after selected PS nanobeads were removed during heat-treatment. Selenization of precursor metal salts and carbonization of poly(vinyl alcohol) (PVA) occurred concurrently during heat-treatment, leading to the expectation that the PZCN could release severe mechanical stress/strain during cycling, introduce more contact areas between the active materials and the electrolyte, and provide more active sites during cycling. In this study, the PZCN synthesis mechanism was examined in detail, and the electrochemical performance of the composite nanofibers, as anodes for SIBs, was compared with that of bare ZnSe/CoSe₂ powders.

## 2. Materials and Methods

### 2.1. Sample Preparation

Porous ZnSe/CoSe_2_/C composite nanofibers (PZCN) with uniformly distributed pores were prepared by electrospinning and subsequent selenization. Electrospinning was conducted using a solution containing poly(vinyl alcohol) (PVA, Kanto, PVA 2000), zinc acetate (Zn(CH₃COO)₂∙2H₂O, Junsei, 99%), cobalt acetate (Co(CH₃COO)₂∙4H₂O, Daejung, 98%), and 100 nm polystyrene (PS) nanobeads in ethanol (C₂H₅OH, Duksan, 99.9%). The size-controlled PS nanobeads were prepared by an emulsion polymerization method previously described [17]. The electrospinning process solution was prepared by adding 1.2 g of PVA, 0.5 g of zinc acetate, 1.135 g of cobalt acetate, and 10 mL of ethanol to 15 mL of PS nanobeads suspension. After vigorous stirring overnight, the electrospinning process was conducted under the following specific conditions: 0.5 mL h^−1^ flow rate, 15 cm distance between the tip and collector, 20 kV applied voltage, and 300 rpm drum rotation rate.

The as-spun nanofibers were stabilized at 160 °C for one day. The stabilized nanofibers were loaded with selenium metal powder (Se, Samchun, 99.5%) into an alumina boat and then selenized, at 400 °C for 3 h, with a ramping rate of 1 °C min^−1^, under a 5% H₂/Ar mixed gas atmosphere. During the selenization process, H₂ gas reacted with the Se metal powder and formed H₂Se gas, which converted the precursor nanofibers into PZCN.

Bare ZnSe/CoSe₂ powders were also prepared for comparison. For this, the as-spun nanofibers were heat-treated at 400 °C under an air atmosphere in order to obtain bare ZnCo₂O₄ nanofibers without carbon. Eventually, bare ZnSe/CoSe₂ powders were obtained after selenization of the bare ZnCo₂O₄ nanofibers at 400 °C for 3 h in H₂Se gas.

### 2.2. Characterization Techniques

The crystal structures of the samples were investigated by X-ray diffraction (XRD, Bruker AXS, D8 Discover with GADDS, Billerica, MA, USA). The morphologies of the samples were observed by field-emission scanning electron microscopy (FE-SEM, Zeiss, ULTRA PLUS, Oberkochen, Germany) and field-emission transmission electron microscopy (HR-TEM, JEOL, JEM-2100F, Tokyo, Japan) at working voltages of 3 kV and 200 kV, respectively. X-ray photoelectron spectroscopy (XPS, Thermo Scientific, K-Alpha, Waltham, MA, USA) was performed on the samples, using Al–K_α_ radiation (1486.6 eV). The characteristics of the carbonaceous materials in the PZCN were analyzed by Raman spectroscopy (LabRam, HR800, Horiba Jobin-Yvon, Paris, France, excitation source = 515 nm Diode laser), at room temperature. Thermogravimetric analysis (TGA, SDT Q600, New Castle, DE, USA) was performed in air, at a heating rate of 10 °C min^−1^. The carbon content of PZCN was analyzed with an elemental analyzer (EA, Vario MICRO cube, Elementar, Langenselbold, Germany), and the sample surface areas were determined using the Brunauer–Emmett–Teller (BET) method, where N₂ was the adsorbate gas.

### 2.3. Electrochemical Measurements

Sample electrochemical properties were analyzed by constructing a 2032-type coin cell. The working electrode was prepared by mixing the active material, carbon black, and sodium carboxymethyl cellulose (CMC) in a 7:2:1 weight ratio. Na metal and a microporous polypropylene film were used as the counter electrode and separator, respectively. In this study, carbonate-based and ether-based electrolytes were used for cell assembly. The carbonate-based electrolyte was prepared by dissolving 1 M NaClO₄ and 5% fluoroethylene carbonate (FEC) in a mixture of 1:1 v/v ratio ethylene carbonate (EC) and dimethyl carbonate (DMC). The ether-based electrolyte was prepared by dissolving 1 M of sodium trifluoromethanesulfonate (NaCF₃SO₃) in diethylene glycol dimethyl ether (DEGDME). Discharge–charge characteristics of the samples were investigated by cycling over a potential range of 0.001–3.0 V, for the carbonate-based electrolyte, and 0.3–2.9 V for the ether-based electrolyte, at various current densities. Cyclic voltammetry (CV) curves were measured, at a scan rate of 0.1 mV s^−1^. The working electrode containing the samples was 1.4 cm × 1.4 cm, and the mass loading was approximately 1.2 mg cm^−2^. In this study, the capacities of the samples were calculated based on the total mass of the prepared sample. Electrochemical impedance spectra were obtained by performing alternating-current electrochemical impedance spectroscopy (EIS, ZIVE SP1), over a frequency range of 0.01 Hz to 100 kHz.

## 3. Results and Discussion

**Figure 1:**
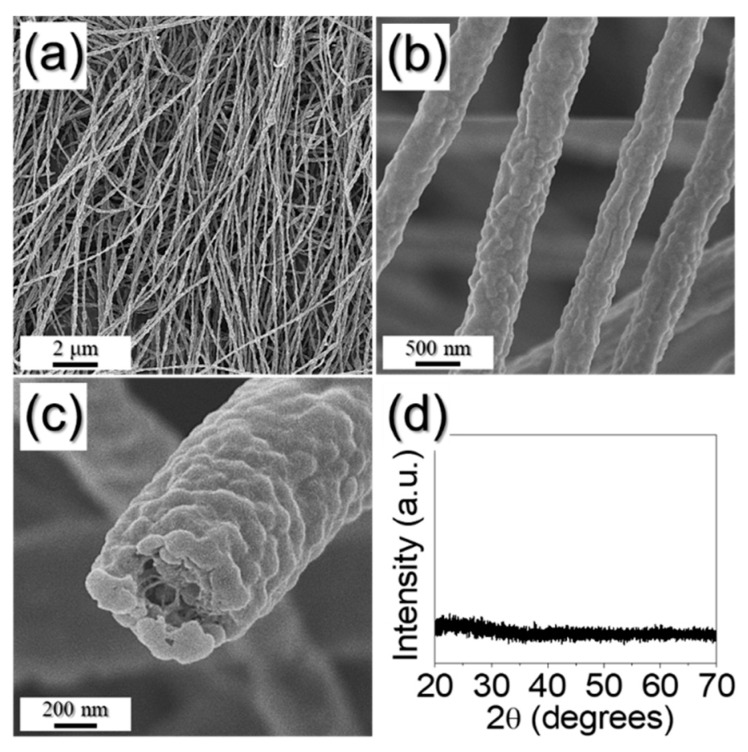
Morphologies, XRD pattern of as-spun nanofiber: (**a**–**c**) FE-SEM images; and (**d**) XRD pattern.

Porous ZnSe/CoSe_2_/C composite nanofibers (PZCN) with uniformly distributed pores were prepared by the electrospinning process and subsequent simple selenization treatment of the as-spun fibers. As a soft template for the formation of meso- and macropores, size-controlled, 100 nm PS nanobeads, prepared by emulsion polymerization, were added in the precursor spinning solution. The PS nanobead synthesis process has been described in detail previously [17]. Even though the PS nanobeads were mixed in a spinning solution with PVA, Zn and Co salts, a stable jet was formed during the electrospinning process, allowing a uniform 1-D nanostructure to be obtained via the electrospinning process, as shown in Figure 1a.

The nanofibers’ embossed fiber surfaces, as shown in Figure 1b,c, were due to the PS nanobeads in their structures, and their homogeneous thickness (580 nm) evidenced the uniform distribution of PS nanobeads in the as-spun nanofibers. From the fractured FE-SEM image in Figure 1c, the pores were observed as the PS nanobeads physically fell from the structure when the nanofiber was broken. It was also hard to identify any phase from the XRD result before heat-treatment, as shown in Figure 1d.

**Figure 2:**
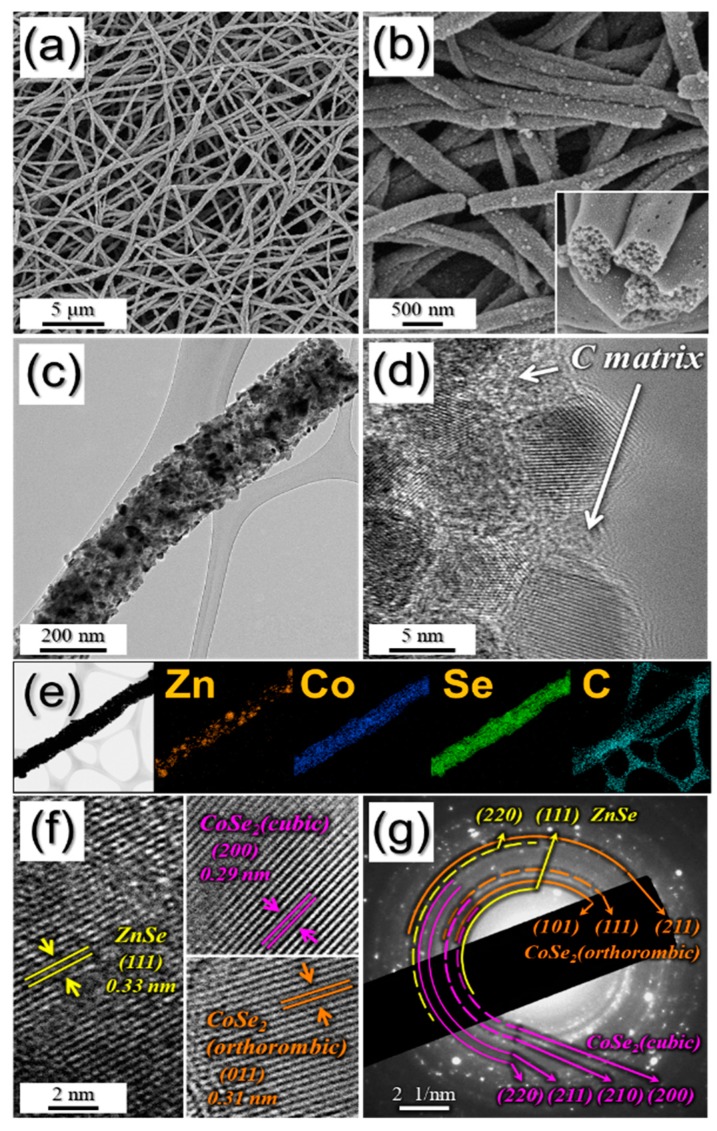
Morphologies, SAED pattern, and elemental mapping images of porous ZnSe/CoSe_2_/C composite nanofibers: (**a**,**b**) FE-SEM images; (**c**) TEM image; (**d**) HR-TEM image; (**e**) elemental mapping images; (**f**) HR lattice images; and (**g**) SAED pattern.

PZCN with uniformly distributed pores obtained after 400 °C heat-treatment of as-spun nanofibers, can be seen in Figure 2. Even after heat-treatment, the one-dimensional nanostructure was well maintained, as shown in Figure 2a. However, the embossed and smooth surface of the as-spun nanofibers was changed into a rough surface, as numerous 26 nm nanoparticles were formed, after heat-treatment, as shown in Figure 2b,c. In addition, the PS nanobeads used as a template in the composite were decomposed into gas, which generated uniformly distributed meso- and macropores with mean size of 50 nm in the structure, after shrinkage during the heat-treatment, as shown in the inset image in Figure 2b. More detailed structures after heat-treatment were confirmed from TEM images (Figure 2c,d). During heat-treatment, PVA in the as-spun fibers was carbonized, therefore a carbon matrix was formed in the structure, and this surrounded the newly formed nanoparticles, as shown in Figure 2d. The elemental mapping images shown in Figure 2e reveal uniform distributions of Co, Se, and C components, and not Zn, over the entire structure. During the selenization process, Zn metal, with its lower melting point (419.5 °C) compared to that of Co metal (1495 °C) was phase-separated from other elements, which caused the separation of ZnSe in the composite [18]. From the high-resolution nanoparticle HR-TEM image in Figure 2f, it can be seen that the lattice fringes were clearly separated, by 0.33, 0.29, and 0.31 nm, which correspond to the (111) lattice plane of cubic ZnSe, the (200) lattice plane of cubic CoSe₂, and the (011) lattice plane of orthorhombic CoSe₂. These crystal phases were confirmed further by the selected area electron diffraction (SAED) pattern in Figure 2g.

**Figure 3:**
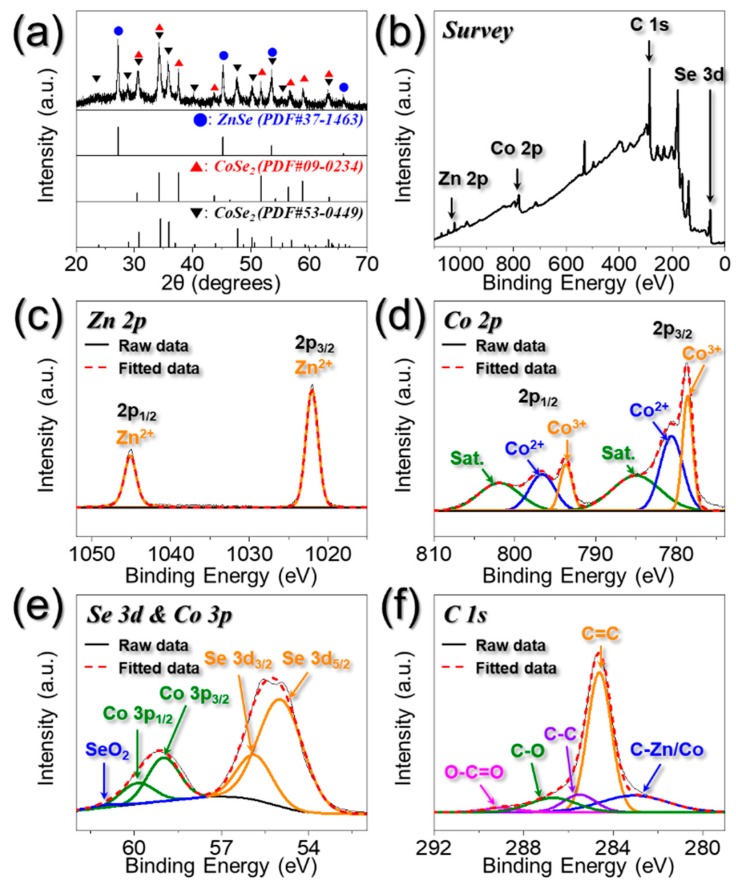
XRD pattern and XPS spectra of porous ZnSe/CoSe_2_/C composite nanofibers: (**a**) XRD pattern; (**b**) wide-scan XPS spectrum; (**c**) Zn 2p XPS spectrum; (**d**) Co 2p XPS spectrum; (**e**) Se 3d and Co 3p XPS spectrum; and (**f**) C 1s XPS spectrum.

**Figure 4:**
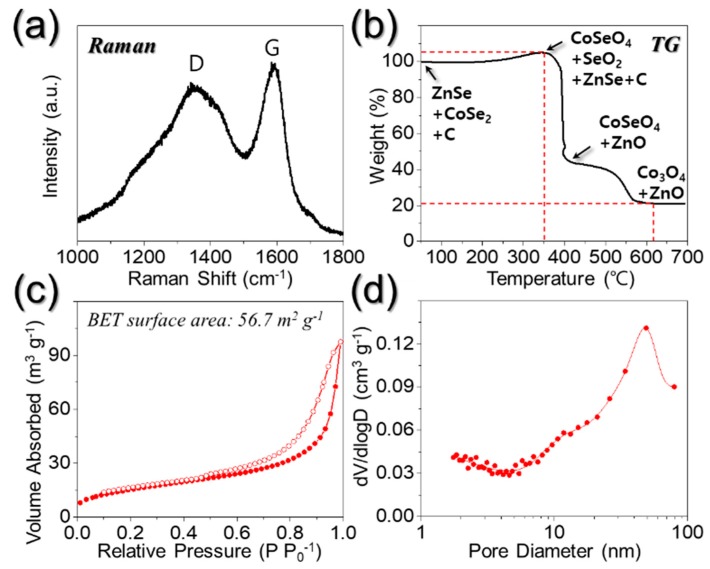
(**a**) Raman spectrum; (**b**) TG curve; (**c**) N_2_ gas adsorption and desorption isotherm; and (**d**) Barrett-Joyner-Halenda (BJH) pore size distribution of porous ZnSe/CoSe_2_/C composite nanofibers.

The characteristics of the resulting PZCN obtained after heat-treatment are shown in Figure 3 and Figure 4. The XRD result shown in Figure 3a proves that the porous nanofibers were composed of one ZnSe and two CoSe₂ crystal phases, which was in line with the SAED patterns (Figure 2g). Therefore, nano-sized ZnSe and CoSe₂ particles, several tens of nanometers in size, were distributed uniformly, along with carbon matrix, over the whole, 1-D nanostructure. Results from XPS analyses of PZCN chemical states can be seen in Figure 3b–f, with the XPS survey spectrum (Figure 3b) confirming the presence of Zn, Co, Se, and C. The Zn 2p spectrum, in Figure 3c, exhibited two obvious peaks, located at 1022.0 and 1045.0 eV, corresponding to Zn 2p_3/2_ and Zn 2p_1/2_ of ZnSe, respectively [19,20]. As shown in Figure 3d, the Co 2p spectrum showed each of the deconvoluted three peaks in the two doublet peaks, Co 2p_3/2_ and Co 2p_1/2_. Co^2+^ peaks, located at 780.6 and 796.6 eV, were attributed to CoSe₂ [21,22]. The existence of the Co^3+^ peaks in the Co 2p spectrum was due to the partial surface oxidation of PZCN under air atmosphere [23,24]. Additionally, shake-up satellites at higher-energy were caused by the antibonding orbital between selenium and cobalt atoms. The Se 3d spectrum (Figure 3e) showed two deconvoluted peaks, at 55.0 and 55.9 eV, corresponding to Se 3d_5/2_ and Se 3d_3/2_ for metal selenides, respectively [19,20,21,22]. The peaks located within 58–61 eV originated from Co 3p and Se–O bonding, attributed to metal selenide partial surface oxidation. The C 1s spectrum in Figure 3f comprised peaks corresponding to C–metal, C=C, C−C, C–O, and O–C=O bonding, at 283.1, 284.6, 285.5, 286.7, and 289.0 eV, respectively [25,26,27]. The peak corresponding to the C=C bond exhibited high intensity, whereas that associated with the C=O bond showed low intensity. This indicated that graphitic carbon was formed through the graphitization mechanism, during the selenization process.

The carbon matrix of PZCN was characterized by means of Raman spectroscopy, as shown in Figure 4a. The degree of graphitization of carbon can typically be evaluated according to the intensity ratio of the D and G bands of carbon at approximately 1350 and 1590 cm^−1^, respectively [28,29]. The peak intensity ratio between the D and G bands (I_D_/I_G_) for PZCN was approximately 0.9. The TG curve of PZCN can be seen in Figure 4b. The first weight increase, starting from ~250 °C, resulted from CoSe_2_ crystal decomposition into CoSeO_4_, and SeO_2_ [30]. The following steep weight loss, at ~350 °C, was due to carbon combustion, oxidation from ZnSe into ZnO, and SeO_2_ vaporization [30,31]. The sample weight loss was slightly diminished, however, by CoSe₂ oxidation, into CoSeO_4_ and SeO_2_. The final weight decrease between 400 and 600 °C was attributed to both the further decomposition of CoSeO_4_ into Co_3_O_4_, and the continuous SeO₂ vaporization in the composite [30]. Based on TG and elemental analysis (EA), as documented in Table S1, the carbon content in the PZCN was approximately 15 wt%. The Brunauer–Emmett–Teller (BET) surface area of PZCN was calculated as 56.7 m^2^ g^−1^, as shown in Figure 4c, with this high BET value caused by the presence of micropores in the carbon matrix decomposed from PVA, and meso- and macropores formed by PS nanobeads. The Barrett–Joyner–Halenda (BJH) pore size distributions of PZCN, as reproduced in Figure 4d, showed micropores with diameters <2 nm, and meso- and macropores with diameters between 50 and 100 nm, which were attributed to pores formed by PS nanobead decomposition.

**Figure 5:**
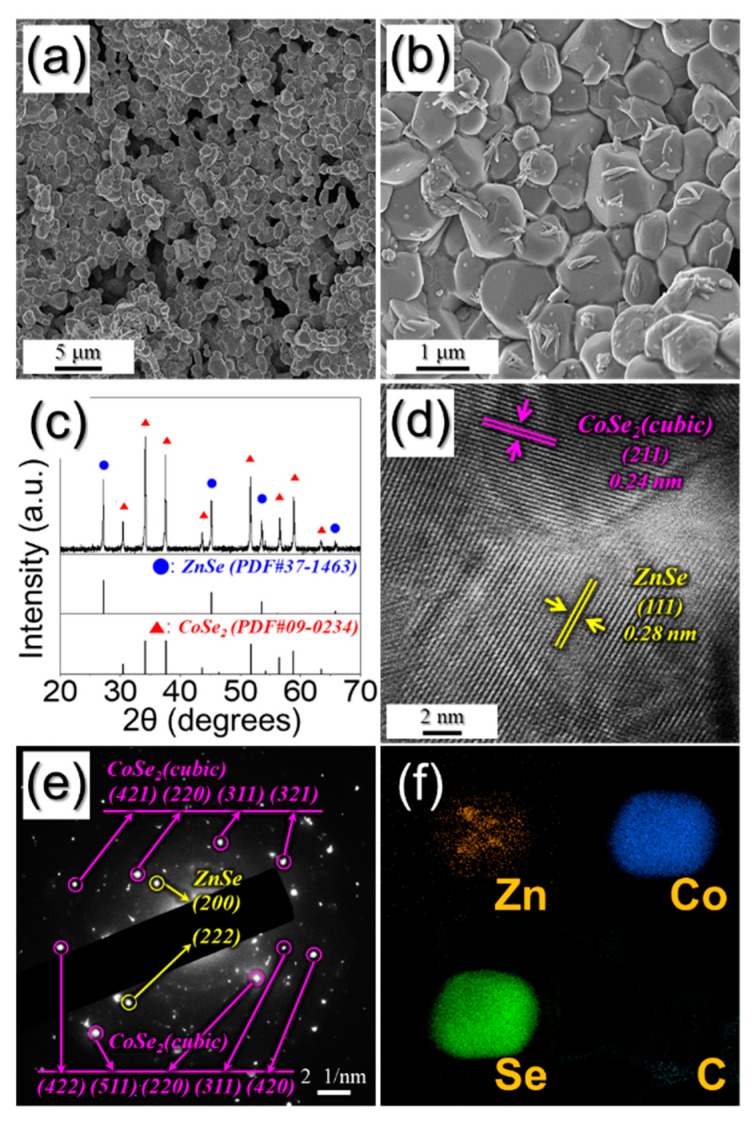
Morphologies, SAED pattern, and elemental mapping images of bare ZnSe/CoSe_2_ powders: (**a**,**b**) FE-SEM images; (**c**) XRD pattern; (**d**) HR lattice image; (**e**) SAED pattern; and (**f**) elemental mapping images.

To prove the structural merits of PZCN as anodes for Na ion storage properties, bare ZnSe/CoSe₂ powders were also prepared. For this, ZnCo₂O₄ nanofibers, which were obtained by oxidation of as-spun fibers, at 400 °C under air (Figure S1), were subsequently selenized, at 400 °C in an H_2_/Ar atmosphere. After selenization, the 1-D nanostructure collapsed, and then aggregated into a powder structure with a mean size of 1.0 μm, as shown in Figure 5a,b. The resulting powders were composed with ZnSe and CoSe₂ phases, as shown in Figure 5c. The lattice fringes in the high-resolution TEM image (Figure 5d) and SAED pattern (Figure 5e) proved the cubic ZnSe and cubic CoSe₂ compositions. The elemental mapping images shown in Figure 5f reveal the uniform distribution of Co and Se components without carbon—which had been completely decomposed by oxidation before the selenization. In addition, the Zn component was partially separated in the structure, as a ZnSe phase.

**Figure 6:**
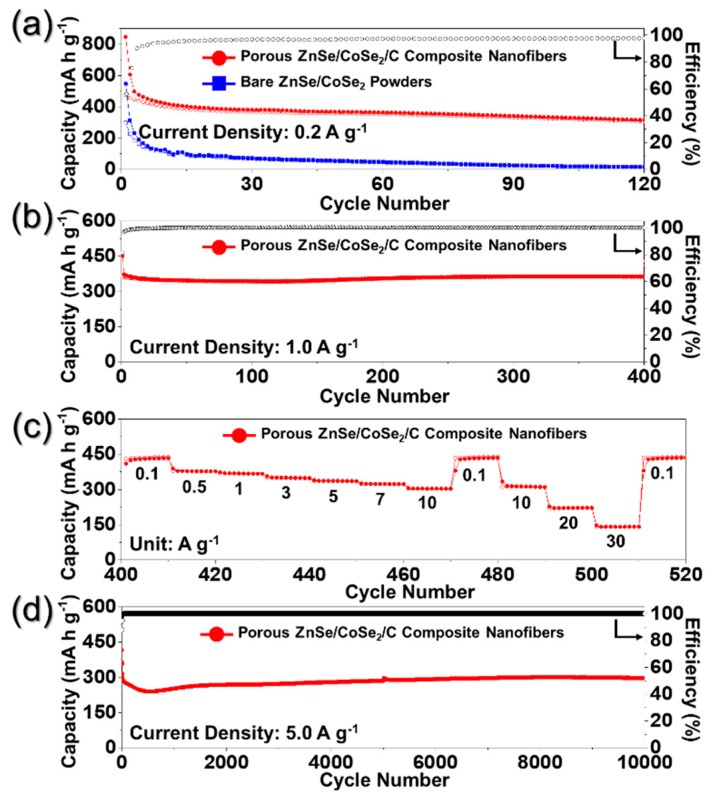
Electrochemical performances of the porous ZnSe/CoSe_2_/C composite nanofibers and bare ZnSe/CoSe_2_ powders: (**a**) cycling performances and Coulombic efficiencies of the cell assembled with carbonate-based electrolyte; (**b**) cycling performance; (**c**) rate performance; and (**d**) long-term cycling performance of the porous ZnSe/CoSe_2_/C composite nanofibers assembled with ether-based electrolyte.

PZCN and bare ZnSe/CoSe₂ powder electrochemical performance, for use in Na-ion storage applications, were compared (Figure 6). The cyclic voltammetry (CV) curves of PZCN and bare ZnSe/CoSe_2_ powders performed at a scan rate of 0.1 mV s^−1^ over the voltage range of 0.3–2.9 V for five cycles can be seen in Figure S2a,b. In the first cathodic scan, the samples both showed main peaks at ~1.0 and 0.3 V. The peak at 1.0 V was attributed to the formation of solid-electrolyte interface (SEI) layer, based on electrolyte decomposition and conversion reaction of CoSe_2_ into Co and Na_2_Se [22,32]. The other peak, at 0.3 V, was due to the conversion of ZnSe into Zn and Na_2_Se [20,33]. In the first anodic scan, both samples exhibited four peaks, at 1.0, 1.5, 1.8, and 1.9 V. The peak at around 1.0 V was derived in restoration to ZnSe, the small peak at 1.5 V was attributed to the formation of Na_x_CoSe₂, while the peaks at ~1.8 and 1.9 V represented the recovery to CoSe_2_ [20,22,32,33]. From the second cycle onward, four new cathodic peaks were seen, at 1.5, 1.1, 0.8, and 0.6 V. The peak at 1.5 V was due to the formation of Na_x_CoSe_2_, and the peak at 1.1 V was due to the formation of CoSe and Na_2_Se from the reaction of Na_x_CoSe_2_ and Na ion [22,32]. The peak at 0.8 V was due to the conversion reaction of CoSe into Co and Na_2_Se, while the peak at 0.6 V was from the conversion reaction of ZnSe into Zn and Na₂Se [20,22,32,33]. Interestingly, the gradual growth of a small peak at 1.5 V (Figure S3c,d)—which was due to the activation of active materials—was observed [22,32].

The cycle performances of the PZCN and bare ZnSe/CoSe_2_ powders were investigated at a current density of 0.2 A g^−1^, by assembling the cell with a carbonate-based electrolyte (Figure 6a). Compared with bare ZnSe/CoSe₂ powders, PZCN showed better cycling properties, up to 120 cycles. The carbon matrix enclosing the active materials and the uniformly distributed meso- and macropores could have acted as buffers in accommodating the volume variations of ZnSe and CoSe_2_ during repeated cycling. The first discharge and charge capacities of PZCN were 846 and 475 mA h g^−1^, respectively, and their corresponding Coulombic efficiency (CE) was 56%. In contrast, bare ZnSe/CoSe₂ powder showed relatively poor cycling properties, as shown in Figure 6a. The first discharge and charge capacities of the bare ZnSe/CoSe_2_ powders were 546 and 299 mA h g^−1^, respectively, and their corresponding CE was 55%. The amount of active material in PZCN per unit is less than that of the bare sample. This indicates that the initial reversible specific capacity of the bare sample should be higher than that of the composite nanofibers, however, the polarization caused by slow Na ion diffusion resulted in the low initial reversible specific capacity of the bare ZnSe/CoSe_2_ powders. In addition, it could be seen that bare ZnSe/CoSe_2_ powder capacity continuously faded during 120 cycles, and this rapid capacity fading was ascribed to pulverization of the electrode by the huge active material volume changes that took place during the repeated discharge–charge processes. The cycling property of PZCN was also investigated in voltage windows between 0.3 and 2.9 V, using the ether-based electrolytes, with the results shown in Figure 6b. It has been reported that, in general, ether-based electrolytes have a low activation energy barrier for Na ion diffusion on the structure, and high stability during repeated cycles, which contribute to the good electrochemical properties of the cells, when assembled for SIBs with metal selenide anodes [34,35,36]. The cycling performance and initial CE of PZCN were improved, even at a higher current density of 1.0 A g^−1^. The first discharge capacity and CE were 451 mA h g^−1^ and 81%, respectively, and the discharge capacity at the 400th cycle was 363 mA h g^−1^, indicating that the capacity retention measured from the second cycle was 97%.

The rate performance of PZCN after 400 cycles is shown in Figure 6c, with the current density increasing from 0.1 to 30.0 A g^−1^, and with 10 cycles performed at each step, and it can be seen that PZCN exhibited excellent rate performance. The uniquely structured nanofibers, with their many meso- and macropores, decreased the Na ion diffusion distance and increased their diffusion rate. What is more, the carbon matrix surrounding the active nanomaterials, which has higher electrical conductivity, enhanced the rate property of PZCN. The final rate capacities of PZCN were 438, 377, 367, 348, 335, 323, and 303 mA h g^−1^ at current densities of 0.1, 0.5, 1, 3, 5, 7, and 10 A g^−1^, respectively. When the current density returned to 0.1 A g^−1^, the discharge capacity of the sample recovered well, to 439 mA h g^−1^. Subsequently, at the higher current densities of 10, 20, and 30 A g^−1^, respectively, the final rate capacities were 310, 222, and 141 mA h g^−1^. Even at the extremely high current density of 30.0 A g^−1^, the PZCN delivered a high discharge capacity, thus proving their high level of stability. The long-term cycling performance of PZCN at the high current density of 5.0 A g^−1^ can be seen in Figure 6d. During the initial several cycles, the discharge capacities were slightly decreased, from 365 mA h g^−1^ at the second cycle to 239 mA h g^−1^ at the 500th cycle. This initial capacity loss of PZCN was attributed to the partial destruction of the internal structure, the irreversible electrochemical decomposition of the electrolyte, and the subsequent formation of a SEI layer on the surface. However, the discharge capacities were stable up to 10,000 cycles. The discharge capacity of PZCN at 10,000th cycle was 297 mA h g^−1^ and the capacity retention measured from the second cycle was 81%.

**Figure 7:**
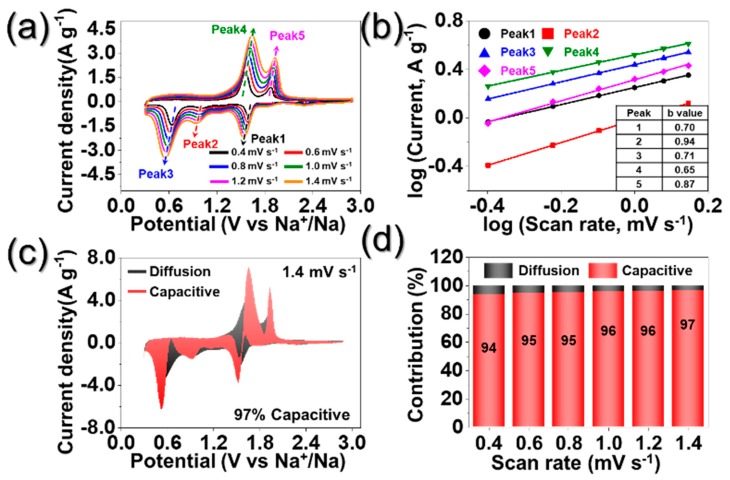
Kinetics investigation: (**a**) CV curves at different scan rates; (**b**) corresponding log(*i*) vs. log(*v*) plots at each redox peak; (**c**) CV curve with the pseudocapacitive proportion at a scan rate of 1.4 mV s^−1^; and (**d**) bar chart exhibiting the percent of pseudocapacitive contribution at different scan rates; (*i*: peak current, *v*: scan rate).

To investigate the excellent rate performance of PZCN, CVs obtained at various scan rates, in voltage windows between 0.3 and 2.9 V, can be seen in Figure 7a. In general, the capacitive contribution of the cell was characterized by analyzing the measured current (*i*) at various scan rates (*v*), according to the following relationship [37,38]:

*i = av^b^* (1)

*log (i) = blog (v) + log (a)* (2)

*I (V) = k_1_v + k_2_v^1/2^* (3)

When the value of *b* is close to 0.5 and 1.0, the electrochemical process is dominated by diffusion-controlled and pseudocapacitive behaviors, respectively [37,38]. Figure 7b shows the linear relationship between log (*i*) and log (*v*), where the slope is defined as value of *b*. The *b* values of porous ZnSe/CoSe_2_/C composite nanofibers were close to 1.0, which proved superior rate performance of PZCN. The proportions of the capacity-contributed pseudocapacitive, at various scan rates were quantitatively analyzed using Equation (3), where *k_1_v* and *k_2_v*^1/2^ correspond to pseudocapacitive and diffusion-controlled contributions, respectively. The constant *k_1_* and *k_2_* value are determined by the slope and intercept from plotting *i*(V)/*v*^1/2^ vs. *v*^1/2^, respectively, at each potential. The pseudocapacitive contribution, at the scan rate of 1.4 mV s^−1^, can be seen in Figure 7c and occupied an area of 97%. The pseudocapacitive contributions at various scan rates can be seen in Figure 7d and revealed higher contributions at higher scan rates. Even at the low scan rate of 0.4 mV s^−1^, the pseudocapacitive contribution of PZCN was as high as 94%. These results demonstrate the excellent rate performance of PZCN.

**Figure 8:**
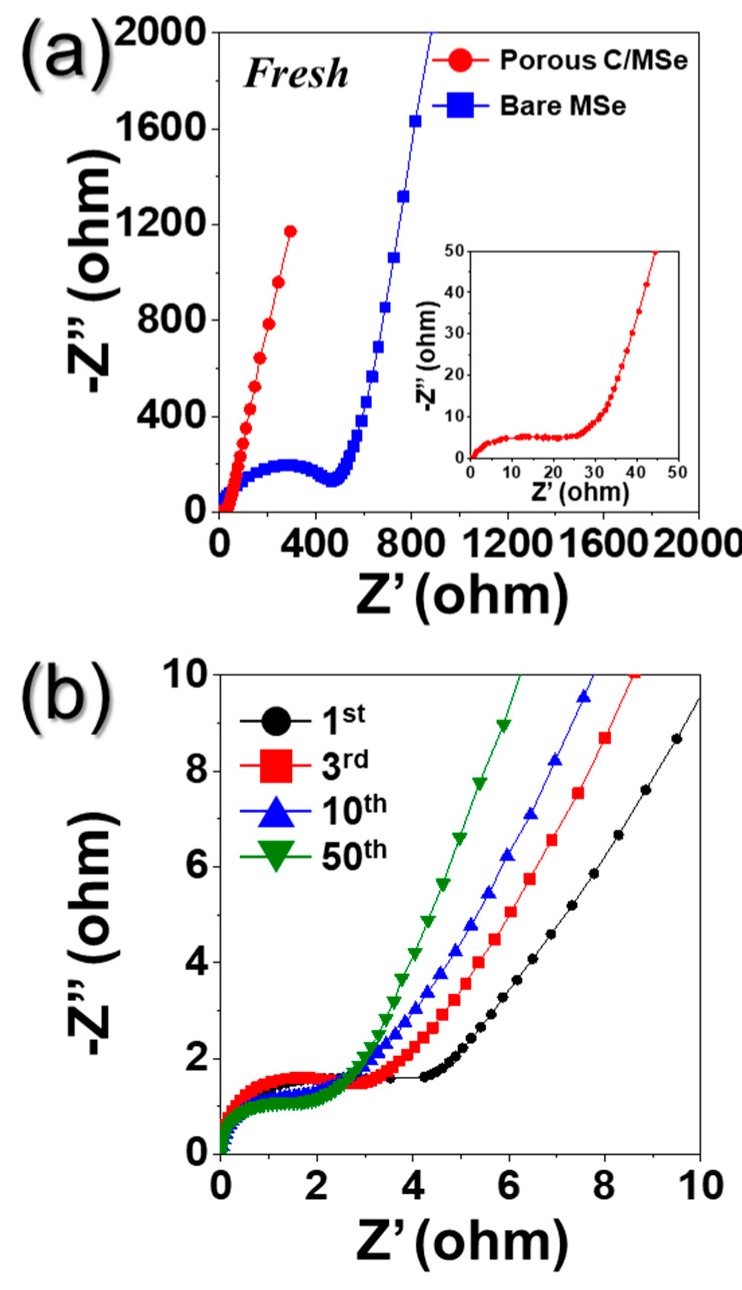
Nyquist plots of the porous ZnSe/CoSe_2_/C composite nanofibers and bare ZnSe/CoSe_2_ powders: (**a**) before cycling; and (**b**) after cycling of porous ZnSe/CoSe_2_/C composite nanofibers.

The superior Na^+^ ion storage properties of PZCN were supported by Electrochemical Impedance Spectroscopy (EIS) analysis, as shown in Figure 8. EIS was carried out on the electrode before and after 1, 3, 10, and 50 cycles with ether-based electrolyte under fully charged state. The semicircles in the medium-frequency range of Nyquist plots were used to measure the charge-transfer resistance (R_ct_) of the electrode [20,23]. The R_ct_ value of PZCN before cycling was ~25 Ω, which is very low compared to that of bare ZnSe/CoSe_2_ powders (~530 Ω) (Figure 8a). The uniform distribution of ZnSe/CoSe_2_ nanoparticles in the C matrix with high electrical conductivity lowered the charge transfer resistance of PZCN. After the first cycle, the R_ct_ value of PZCN decreased ~5 Ω due to the formation of small-sized nanocrystals during cycling [5,23]. In addition, the low R_ct_ values of PZCN were well maintained even after repeated charge and discharge processes, as shown in Figure 8b. This result demonstrates the excellent stability of PZNC for repeated Na^+^ insertion and extraction over cycles (Figure 8b).

**Figure 9:**
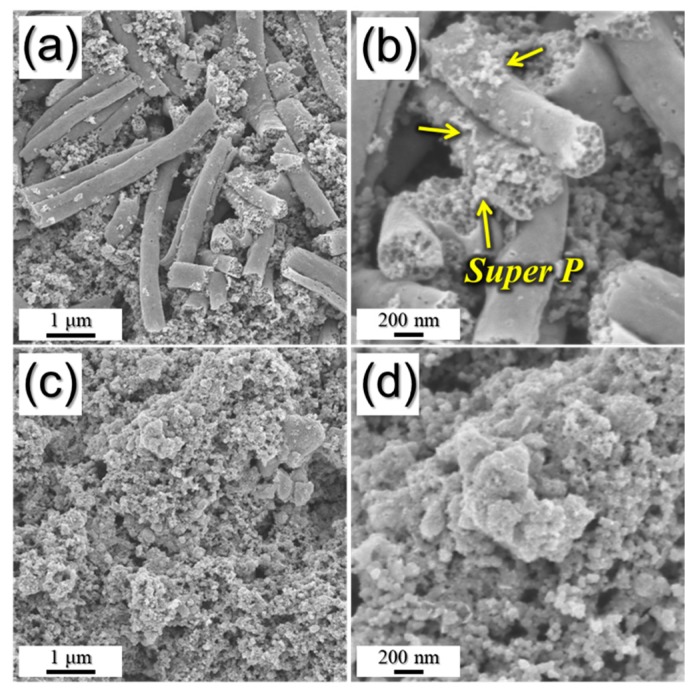
Morphologies of: (**a**,**b**) porous ZnSe/CoSe_2_/C composite nanofibers; and (**c**,**d**) bare ZnSe/CoSe_2_ powders obtained after 120 cycles.

To confirm the structural stability of PZCN, the morphologies of samples after 120 cycles were observed, as shown in Figure 9. The PZCN maintained their original porous structure even after 120 cycles at a current density of 0.2 A g^−1^, as shown in Figure 9a,b. However, bare ZnSe/CoSe_2_ powders had been broken into several pieces after cycling (Figure 9c,d). The large volume variation during the repeated sodium insertion and desertion destroyed the powder structure. These results obviously exhibit the structural robustness of PZCN.

## 4. Conclusions

In this study, porous structured bimetallic ZnSe/CoSe₂/carbon composite nanofibers with uniformly distributed pores, fabricated by electrospinning and subsequent simple heat-treatment processes, were introduced as anodes in sodium-ion batteries. As a soft template for the formation of meso- and macropores, the size-controlled, 100 nm PS nanobeads, prepared by emulsion polymerization, were added to the precursor spinning solution. The PS nanobeads played a key role in the formation and uniform distribution of pores in the nanofiber structure, after they were selectively removed during the heat-treatment process. Additionally, PVA in the as-spun fibers was carbonized during the heat-treatment, which allowed the carbon matrix surrounding the ZnSe/CoSe₂ nanoparticles to form. During cycling, PZCN released severe mechanical stress/strain, introduced more contact areas between the active materials and the electrolyte, and provided more active sites, allowing them to exhibit excellent Na ion storage properties, in comparison to those of ZnSe/CoSe₂ powders.

## Supplementary Materials

The following are available online at https://www.mdpi.com/2079-4991/9/10/1362/s1, Figure S1: (a and b) Morphologies and (c) XRD pattern of the nanofibers obtained after heat-treatment of as-spun nanofiber at 400 °C under air atmosphere, Figure S2: Electrochemical properties of porous ZnSe/CoSe_2_/C composite nanofibers and bare ZnSe/CoSe_2_ powders: (a,c) cyclic voltammetry (CV) curves of porous ZnSe/CoSe_2_/C composite nanofibers, (b,d) CV curves of bare ZnSe/CoSe_2_ powders, and (e) charge-discharge curves of porous ZnSe/CoSe_2_/C composite nanofibers. All cells assembled with ether-based electrolyte, Figure S3: Cycling performance of the porous ZnSe/CoSe_2_/C composite nanofibers assembled with ether-based electrolyte in voltage windows between 0.001 and 3.0 V, Table S1: Elemental analysis result of porous ZnSe/CoSe_2_/C composite nanofibers.
