# Supplementary material for: Porous Hybrid Nanofibers Comprising ZnSe/CoSe₂/Carbon with Uniformly Distributed Pores as Anodes for High-Performance Sodium-Ion Batteries
Source: Nanomaterials (Basel). 2019 Sep 23;9(10):1362. doi: 10.3390/nano9101362 (PMC6835312; doi:10.3390/nano9101362)
Supplement: Supplementary file 1 [file nanomaterials-09-01362-s001.pdf]

Supporting information

Porous Hybrid Nanofibers Comprising $ZnSe/CoSe_2$/Carbon with Uniformly Distributed Pores as Anodes for High-Performance Sodium-Ion Batteries

Sun Young Jeong and Jung Sang Cho *

Department of Engineering Chemistry, Chungbuk National University, Chungbuk 361-763, Korea
* Correspondence: jscho@cbnu.ac.kr; Tel.: +82-43-261-2489

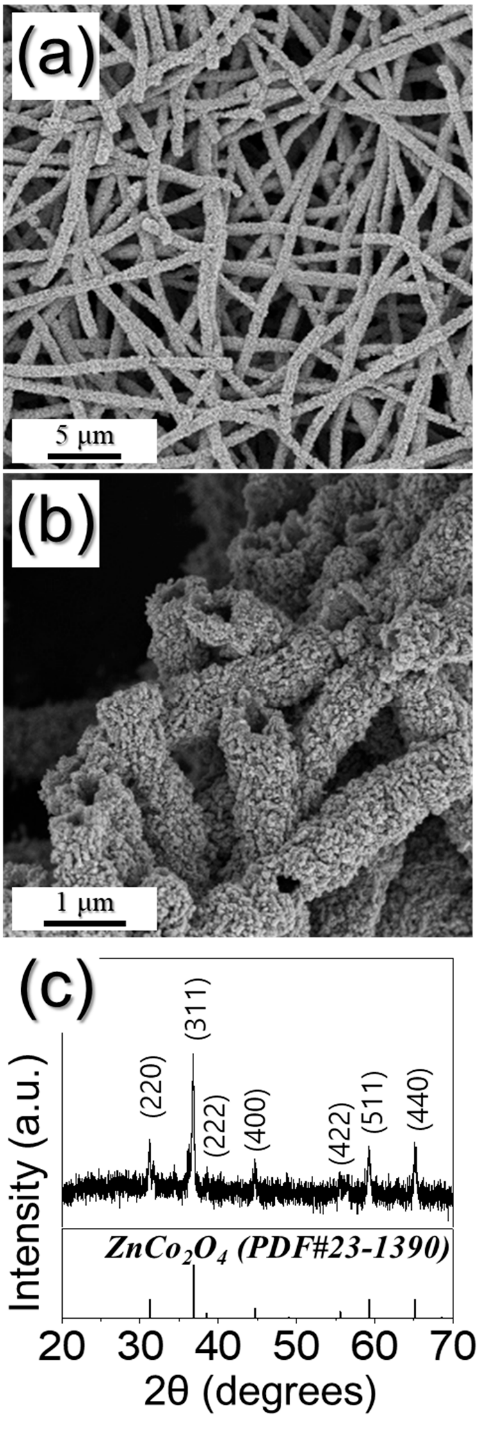

Figure S1. (a and b) Morphologies and (c) XRD pattern of the nanofibers obtained after heat-treatment of as-spun nanofiber at 400℃ under air atmosphere.

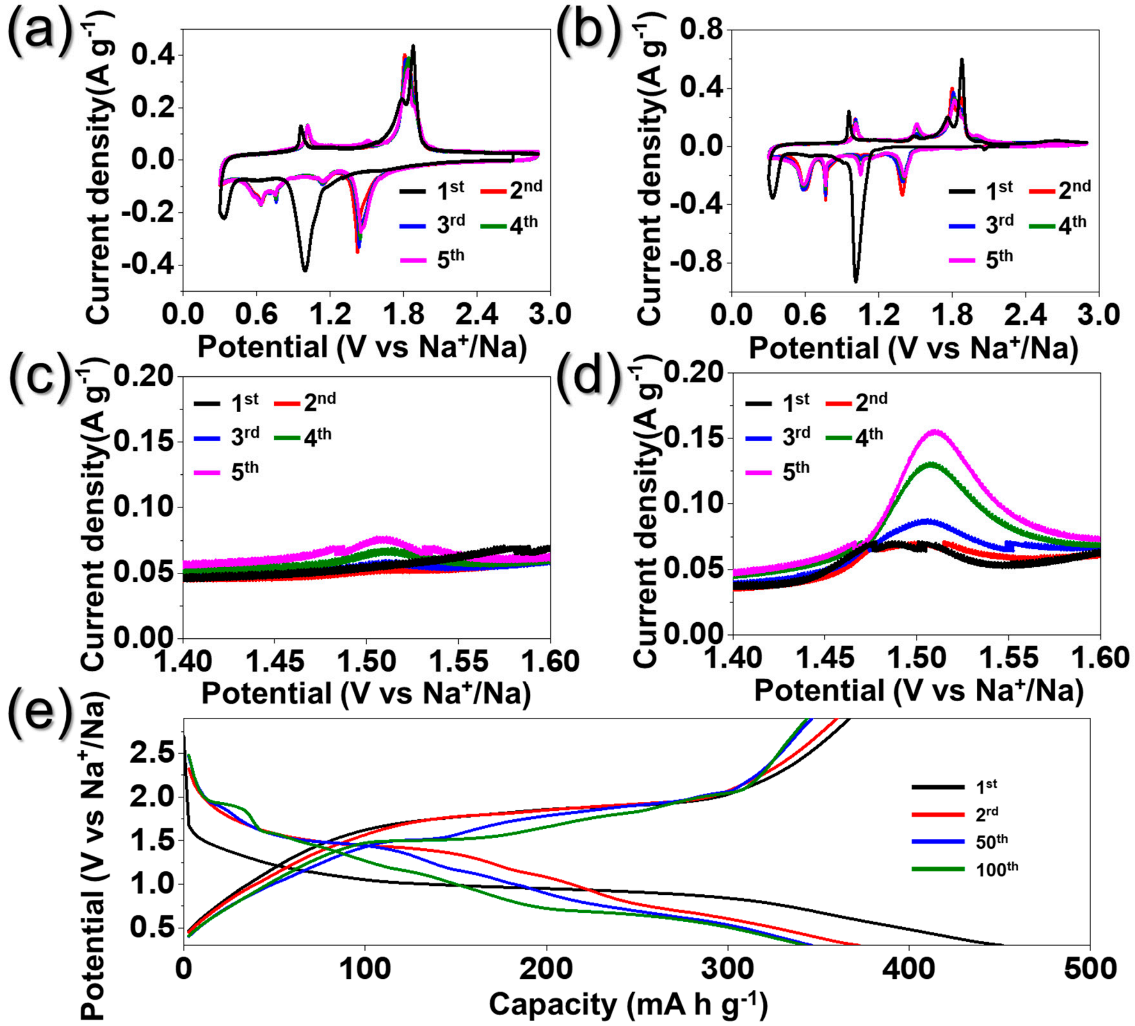

Figure S2. Electrochemical properties of porous $ZnSe/CoSe_2/C$ composite nanofibers and bare $ZnSe/CoSe_2$ powders: (a,c) cyclic voltammetry (CV) curves of porous $ZnSe/CoSe_2/C$ composite nanofibers, (b,d) CV curves of bare $ZnSe/CoSe_2$ powders, and (e) charge-discharge curves of porous $ZnSe/CoSe_2/C$ composite nanofibers. All cells assembled with ether-based electrolyte.

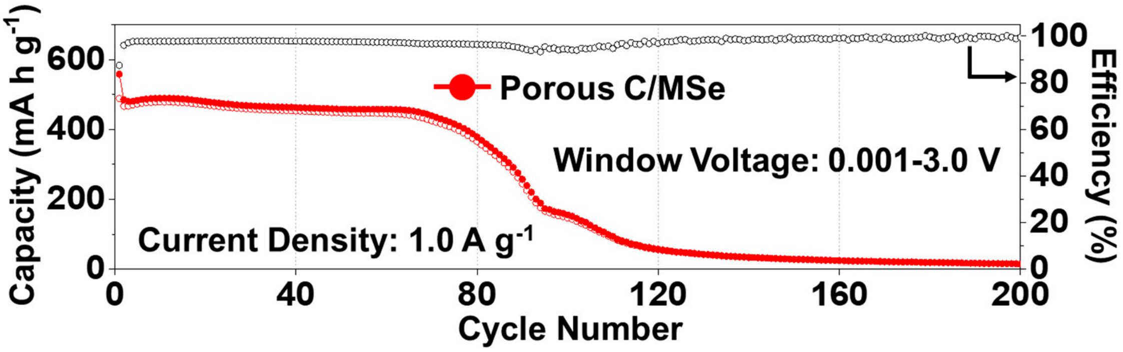

Figure S3. Cycling performance of the porous $ZnSe/CoSe_2/C$ composite nanofibers assembled with ether-based electrolyte in voltage windows between 0.001 and 3.0 V.

Table S1. Elemental analysis result of porous $ZnSe/CoSe_2/C$ composite nanofibers.

Sample	C (wt%)
Sample	15
